# N-acylhydrazone Derivative-Loaded Cellulose Acetate Films: Thermoanalytical, Spectroscopic, Mechanical and Morphological Characterization

**DOI:** 10.3390/polym13142345

**Published:** 2021-07-17

**Authors:** Amaro César Lima de Assis, Lívia Maria Coelho de Carvalho Moreira, Beatriz Patrício Rocha, Milena Raissa Bezerra Pereira, Demis Ferreira de Melo, Ricardo Olímpio de Moura, Eduardo Pereira de Azevedo, João Augusto Oshiro-Junior, Bolívar Ponciano Goulart de Lima Damasceno

**Affiliations:** 1 Graduate Program in Pharmaceutical Sciences, Biological and Health Sciences Center, State University of Paraíba (UEPB), Av. Juvêncio Arruda, s/n, Bairro Universitário, Campina Grande 58429-600, Paraíba, Brazil; amaro_ico@hotmail.com (A.C.L.d.A.); carvalholivia312@gmail.com (L.M.C.d.C.M.); beatrizpattricio@gmail.com (B.P.R.); milena-ray@hotmail.com (M.R.B.P.); demiscz@gmail.com (D.F.d.M.); ricardo.olimpiodemoura@gmail.com (R.O.d.M.); 2 Laboratory of Development and Characterization of Pharmaceutical Products, Department of Pharmacy, Biological and Health Sciences Center, State University of Paraíba (UEPB), Campina Grande 58429-600, Paraíba, Brazil; 3 Department of Pharmacy, State University of Paraiba (UEPB), Campina Grande 58429-600, Paraíba, Brazil; 4 Graduate Program of Biotechnology, Laureate International Universities–Universidade Potiguar (UnP), Natal 59056-000, Rio Grande do Norte, Brazil; azevedoep@hotmail.com

**Keywords:** cellulose acetate, N-acylhydrazonic derivative, thermal analysis, polymeric films

## Abstract

Cellulose acetate (ACT) is one of the most important cellulose derivatives due to its biodegradability and low toxicity, presenting itself as one of the main substitutes for synthetic materials in the development of wound dressing films. The incorporation of a N-acylhydrazonic derivative (JR19), with its promising anti-inflammatory activity, may represent an alternative for the treatment of skin wounds. This work aims to develop and to physicochemically and mechanically characterize ACT films containing JR19. The films were prepared using the ‘casting’ method and further characterized by thermoanalytical and spectroscopic techniques. In addition, mechanical tests and morphological analysis were performed. Thermogravimetry (TG) and differential scanning calorimetry (DSC) analyses showed that the thermal events attributed to excipients and films were similar, indicating the absence of physical incompatibilities between ACT and JR19. Infrared spectroscopy showed that JR19 was incorporated into ACT films. The characteristic band attributed to C≡N (2279 to 2264 cm^−1^) was observed in the spectra of JR19, in that of the physical mixture of JR19/ACT, and, to a lesser extent, in the spectra of JR19 incorporated into the ACT film, suggesting some interaction between JR19 and ACT. X-ray diffraction (XRD) evidenced the suppression of the crystallinity of JR19 (diffraction peaks at 8.54°, 12.80°, 14.09°, 16.08°, 18.19°, 22.65°, 23.59°, 24.53°, 25.70°, 28.16° and 30.27°2θ) after incorporation into ACT films. The mechanical tests indicated the adequate integrity of the films and their resistance to bending. The morphological characterization showed JR19 crystals along with a homogeneously distributed porous structure throughout the surface of the films with an average diameter of 21.34 µm and 22.65 µm of the films alone and of those incorporating JR19F, respectively. This study was able to characterize the ACT films incorporating JR19, showing their potential to be further developed as wound healing dressings.

## 1. Introduction

Polymers are considered the most versatile class of materials, and have been responsible for changing our daily lives in recent decades. The association of polymer science with pharmaceutical sciences has led to the development of several new drug delivery systems. Polymeric delivery systems are primarily intended to achieve temporal or spatial control of drug delivery [1,2].

Cellulose and its derivatives represent a group of natural polymers widely used in pharmaceutical dosage forms, being preferred over synthetic polymers due to their low toxicity, biodegradability, low cost and good availability [3,4]. In addition, their filmogenic characteristics have been an element of high interest and prominence in the group of pharmaceutical excipients [1,5,6].

Cellulose acetate is a cellulose ester that comes in different forms according to its degree of substitution, that is, the number of acetyl groups linked to the hydroxyl groups of a single anhydroglucose unit [7,8]. This polymer has been used to produce a wide variety of products, such as textile products, films for photography, cigarette filters and films for packaging [9]. In addition, Tedeschi et al. [10] reported the use of cellulose acetate for different applications, such as surface coatings, optical films, resins, membranes for separation and to control the release of drugs.

Although this polymer has higher hydrophobicity than cellulose, it is considered to be a hydrophilic and water-absorbent material, with better properties for application in biological systems when compared to common plastic materials derived from oil [11,12]. Cellulose acetylation results in lower toxicity, better stability, higher water permeability, higher glass transition temperature and better compatibility with active agents, which enable its use in the pharmaceutical industry [13].

The skin is considered to be the largest organ in the human body, being responsible for different functions such as protection against friction, ultraviolet radiation, water loss and microorganisms entrance [14]. Despite the variety of wound dressings available on the market for wound regeneration, there is no existing product that meets the characteristics of an ideal dressing [15]. Thus, there is a real need for materials that can accelerate the healing process, in addition to providing greater patient safety and compliance [16].

Devi and Dutta [17] investigated the use of polymeric films to treat skin wounds, in which they showed the several advantages that cellulose acetate can bring when associated with other components. Cellulose acetate is able to interact with other molecules by hydrogen-bond and electrostatic interactions [18]. N-acylhydrazonic derivatives have shown great potential for biomedical application due to the presence of -CO-, -NH-, N=CH- fragments in their structure, which allows them to interact with other molecules or receptors through hydrophobic, electrostatic and hydrogen-bond interactions [19,20].

The N-acylhydrazonic derivative JR19 namely N-(1H-indol-3yl) methylene-2-cyanoacetrohydrazide has been investigated for its antioxidant [21], antifungal [22], antimicrobial [23] and anti-inflammatory activities [21,24,25]. Therefore, JR19 seems to be a promising candidate to be incorporated into cellulose acetate films, especially when the pharmacological application is directed towards the skin. JR19 may interact with other molecules through the more electronegative atoms present in its structure, such as oxygen and nitrogen, in addition to the hydrophobic groups of the indole ring that favors hydrophobic interactions [19].

Physicochemical characterization has been shown to be an essential step for the analysis of the microstructure of drug–polymer systems, being part of the quality control process of pharmaceutical products due to its ability to predict incompatibilities and expiration date. In this context, the aim of this work was to develop and characterize cellulose acetate films incorporated with JR19, which may represent a new alternative treatment for various topical inflammatory disorders.

## 2. Materials and Methods

### 2.1. Material

Cellulose acetate was purchased from Sigma-Aldrich^®^ (San Luis, MO, USA) and identified by the reference number 419028-500G; JR19 compound derived from N-acylhydrazonic was synthesized at the Laboratory of Drug Synthesis and Development (LDSF) of the State University of Paraíba. Acetone (Neon^®^, Suzano, SP, Brazil), bi-distilled glycerol (Viafarma^®^, São Paulo, SP, Brazil), ethanol (Neon^®^) and distilled water were the solvents used in this study.

### 2.2. Preparation of Cellulose Acetate Films with and without JR19

The films were prepared using the casting method, which is based on spreading the film precursor solution on a substrate (glass plate). For the preparation of the films without JR19 (herein named blank films, BF), cellulose acetate (ACT) solution was first prepared at a concentration of 1.5% (*w/w*) by dissolving this polymer in acetone under stirring in an ultrasound probe for 2 min followed by 1 min in an ultrasound bath. Bi-distilled glycerol at a concentration of 10% was used as the film plasticizer. A 5 mL amount of the resulting solution was poured into a Petri dish with a diameter of 5.5 cm followed by drying in a refrigerator with a temperature between 2 and 8 °C for a period of 4 h to remove the solvent. On the other hand, films incorporated with JR19 (JR19F) were prepared by dispersing 10 mg of this drug in 5 mL of the cellulose acetate solution, under stirring in an ultrasound probe for 2 min followed by 1 min in an ultrasound bath. Similar to the BF samples, JR19F were dried in a refrigerator (temperature between 2 and 8 °C) for 4 h. All individual films had their macroscopic and mechanical sensory aspects evaluated.

### 2.3. Physicochemical Characterization

#### 2.3.1. Bending Resistance Test

The bending resistance test was performed with the purpose of measuring the flexibility of the films. This test is necessary in order to predict whether a further wound dressing would be comfortable and safe to apply over a wound surface. The bending resistance is determined manually by repeatedly bending the film at the same point until it breaks or is folded up to 300 times [17]. The amount of bending without any break gives the value of its resistance. The analysis was performed in triplicate and the values were reported as average.

#### 2.3.2. Tensile Test

The tensile test was carried out with the purpose of evaluating the deformation (%) and tensile strength (Mpa) of the films. Prior to the test, films dimensions were measured with a digital micrometer of Colante Proof model, 293 series (MITUTOY^®^, Suzano, Brasil). The tensile test was performed on a Universal Testing Machine, model 3366 (INSTRON^®^, Norwood, MA, USA) following the ASTM D882-02 guidelines [26]. The following parameters were used: specimen of type 4 with a distance between the claws of 12 cm; length of 25 mm and width of 4 mm. The analyses were performed in triplicate in order to obtain an average value.

#### 2.3.3. Optical Microscopy (OM)

The bi (2D) and three-dimensional (3D) microstructures of the films were analyzed using a digital optical microscope (KH7700, Hirox^®^, Tokyo, Japan) at magnitudes of 140× and 1120×. Once the 2D and 3D images were acquired, the dimensions of the microstructures were determined with the aid of the ImageJ software (version 1.53e, National Institutes of Health, Bethesda, MD, USA) and the average surface roughness (Ra) was assessed with Gwyddion software (version 2.56).

#### 2.3.4. Scanning Electron Microscopy (SEM)

The morphological aspects of BF and JR19F were assessed through scanning electron microscopy (SEM) using a bench microscope, model VEGA3 (TSCAN^®^, Brno, Czech Republic) with fixed energy of 8 KV. The samples were previously fixed in carbon tape and coated with a thin layer of gold with a coating process of 80 s and an average coating thickness of 10 nm.

#### 2.3.5. Thermal Analysis

##### Differential Scanning Calorimetry (DSC)

DSC curves were obtained in a Differential Scanning Calorimetric module, model 8500 (Perkin Elmer^®^, Boston, MA, USA). Samples of 2.00 ± 0.05 mg were packed in a hermetically sealed aluminum crucible and analyzed at a heating rate of 10 °C min^−^^1^, with a temperature range of 25–400 °C. A nitrogen atmosphere was used with a flow rate of 50 mL min^−^^1^.

##### Thermogravimetry (TG) and Its Derivative (DTG)

Thermogravimetric curves were obtained on a TGA 400 thermogravimetric module (Perkin Elmer^®^, Boston, MA, USA). Samples of 5.00 ± 0.05 mg were packed in alumina crucibles and heated at a temperature range of 25–900 °C at a heating rate of 10 °C min^−1^, under a nitrogen atmosphere with a flow of 50 mL min^−1^.

#### 2.3.6. Fourier Transform Infrared Spectroscopy (FTIR)

Fourier transform infrared (FT-IR) (4000–650 cm^−1^) spectra were obtained using a Spectrum TM 400 FT-IR/FT-NIR spectrometer (Perkin Elmer^®^, Boston, MA, USA), at a scanning speed of 0.2 cm^−1^ and resolution of 4 cm^−1^.

#### 2.3.7. X-ray Diffraction (XRD)

The crystallinity of the samples was determined by X-ray diffraction in the conventional Bragg-Brentano geometry using an XRD-6000 diffractometer (Shimadzu^®^, Kyoto, Japan) with angular scanning of 5° < 2θ < 35°, θ-2θ system, using Cu (kα1) radiation at a step of 0.02° (2θ) and 0.6 s of interval for each sample.

## 3. Results and Discussion

### 3.1. Development of Cellulose Acetate Films with JR19

**Figure 1:**
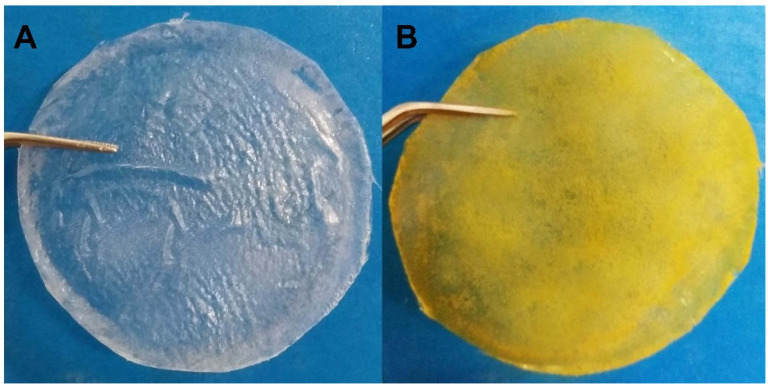
Cellulose acetate films without (**A**) and with JR19 (**B**).

During the initial development of the films, it was observed that concentrations of ACT above 2% resulted in films with poor mechanical properties and inadequate thickness for use as a wound dressing. On the other hand, at a concentration of 0.5% of ACT, the resulting films were fragile and brittle. Thus, films obtained at an ACT concentration of 1.5% showed macroscopically good distribution of the drug and reasonable mechanical sensory properties such as elasticity and flexibility. The BFs were colorless, while the JR19Fs showed an orange color, which is an indication that JR10 was incorporated in the ACT matrix as the color of JR19 is similar to that of the JR19F, as shown in Figure 1.

To improve the mechanical properties of films, plasticizers such as glycerol are usually added to the polymer solution. Therefore, the addition of plasticizer is vital for the formation of strong and elastic films due to their ability to interact with polymer chains, reducing the intermolecular interaction of the polymeric three-dimensional network and providing greater flexibility and resistance. In addition to these effects, plasticizers also play an important role in modulating the drug release from the polymeric films [27,28]. In this current study, the films without plasticizer were fragile and brittle, which led us to use glycerol as plasticizer at a concentration of 10%.

### 3.2. Bending Resistance

The bending resistance test is a simple and straightforward technique that verifies the flexibility of films, and is very important as a tool to predict other mechanical properties, therefore enabling adaptations on the formulation in order to fulfill the requirements for a skin wound dressing [17]. The JR19Fs kept their integrity up to an average of 372 bends, while the BFs showed resistance to an average of 348 bends. These results corroborate the data shown by Wanderley et al. [29], who demonstrated that the presence of JR19 influenced the physicochemical properties of chitosan films, promoting greater resistance to bending the films.

### 3.3. Tensile Test

To investigate the feasibility of films for the development of further wound dressings, their tensile strength and flexibility must be assessed through tensile test [30]. Polymeric films that present low degree of deformation and reasonable values of tensile strength are suggestive of having high potential for wound dressing applications [31,32].

The results of the tensile tests are shown in Table 1. The average thickness of BF and JR19F were 0.132 ± 0.016 mm and 0.137 ± 0.019 mm, respectively, which shows a small increase in the thickness of the films after the incorporation of the drug; however, without statistical significance.

Regarding the tensile strength of BF and JR19F, a slight increase in tensile strength was observed with the incorporation of JR19F, even though this difference in tensile strength was not statistically significant. This result is in accordance with that of the bending test, in which films containing JR19 showed no statistical difference in comparison to those without this drug (BF). Similarly, the incorporation of JR19 resulted in a slight increase in the percentage of deformation, but with no statistically significant difference. The improvement in the mechanical properties of JR19F in comparison to BF may be due to the dispersion of JR19 crystals in the polymer matrix, which may have favored the formation of hydrogen bonds between the dispersed JR19 and ACT, as previously reported by Gao et al. [33] on ACT films containing TiO_2_ dispersed in the polymer matrix.

According to Evans et al. [34], polymeric wound dressings must have tensile strength compatible with that of the skin, which varies from 4 to 30 Mpa. In this current study, BF and JR19F presented tensile strength close to this range, especially when we take into account the standard deviation (Table 1); therefore, we may infer that the films have mechanical properties that are adequate for topical application as wound dressing. Liu et al. [35] and Stachowiak [36] report that the presence of glycerol as plasticizer causes a breakdown of the polymeric network causing an increase in the flexibility of the films and a decrease in tensile strength. Therefore, it seems likely to infer that adjustment in the concentration of the plasticizer might result in improvement in the tensile strength presented by the films.

### 3.4. Optical Microscopy 

**Figure 2:**
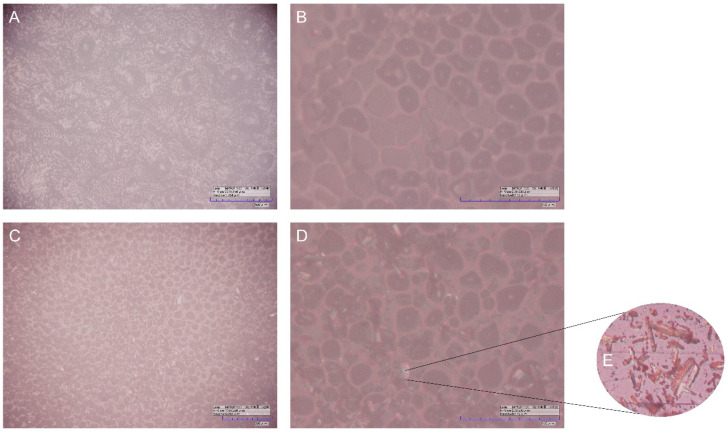
Optical microscopy of BF with magnitude of 280× (**A**) and 1120× (**B**), and that of JR19F with magnitude of 280× (**C**), and 1120× (**D**), as well as crystals of JR19 (**E**).

Optical microscopy was used to investigate the surface structure of BF and JR19, whose micrographs are shown in Figure 2.

The micrographs evidence the presence of small rounded pores homogeneously distributed over the entire surface of the films. These pores had similar dimensions in both films with an average diameter of 21.34 µm and 22.65 µm in the BF and JR19F films, respectively. These pore sizes are higher than those found by Wu, Qin and Li [37], in which ACT films were developed at a concentration of 11% by the casting method and whose pores diameters ranged between 0.127 and 0.300 µm. This difference could be explained by the different concentrations of ACT used in both studies [38], as the ACT films from our study were prepared at a concentration almost 10-fold lower (1.5%). In fact, higher polymeric concentrations favor the ability of the polymer to stabilize a larger surface area of water droplets, in addition to preventing the coalescence of these droplets and, consequently, their growth, resulting in the formation of smaller pores [39,40]. Another factor that is related to this phenomenon is the decrease in the temperature gradient between the polymeric solution and the surrounding atmosphere, where the rapid evaporation of the solvent causes an increase in this gradient. However, more concentrated polymeric solutions cause a decrease in vapor pressure and evaporation speed of the solvent, which favors less condensation of water vapor resulting in the formation of smaller pores [41,42].

The differences between the surface morphology of BF and JR19F are better evidenced in the micrographs taken at magnitude of 1120× (Figure 2B,D), where it is possible to visualize the presence of cylindrical crystals dispersed on the surface of JR19F. Such crystals are similar to those of JR19, which are depicted in Figure 2E. This finding is similar to that presented by Wanderley et al. [29], in which the presence of JR19 crystals in polymeric chitosan films could be visualized. The presence of these crystals in JR19F samples seems to indicate that JR19 was successfully incorporated into the ACT films.

From this finding, the influence of the polymeric matrix on the size of the dispersed crystals was evaluated. The average size of the crystals in JR19F was 11.17 µm, which are smaller than those of JR19 (15.84 µm). This reduction in the crystals size leads to an increase in the surface area of this drug dispersed in the polymeric matrix, which can positively influence the solubility parameter [43,44].

The Ra data showed differences between BF and JR19F, with values of 41.87 and 44.23 µm, respectively. This increase in Ra of JR19F in relation to BF may be directly related to the increase in pore size, as found by Rahimpour et al. [45]. These values follow the same trend shown by Wanderley et al. [29], in which chitosan films with JR19 showed higher Ra values (9.9 µm) compared to chitosan films without this drug (3.71 µm).

**Figure 3:**
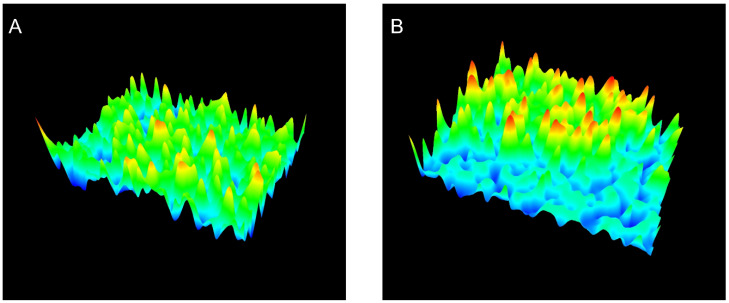
3D images of BF (**A**) and JR19F (**B**).

This increase in Ra for JR19F together with the reduction in crystal size may favor the solubility of JR19 in aqueous media, as the increase in Ra is directly related to the increase in the hydrophilic character of a material [46,47]. The 3D images of the films (Figure 3) confirm this difference in the surfaces between BF and JR19F, as evidenced by the greater number of valleys in the JR19F (Figure 3B) in relation to BF (Figure 3A), which is in accordance to the findings reported by Wanderley et al. [29].

### 3.5. Scanning Electron Microscopy (SEM)

**Figure 4:**
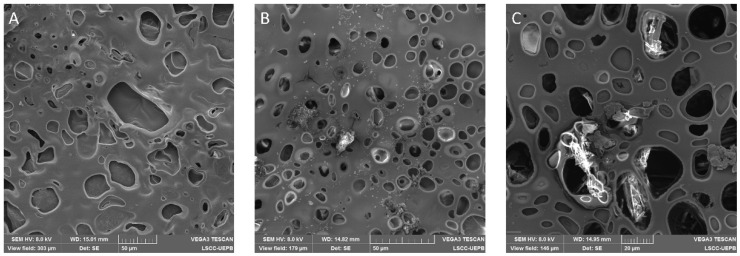
SEM micrographs of BF and JR19F. (**A**) BF (scale of 50 µm); (**B**) JR19F (scale of 50 µm); (**C**) JR19F (scale of 20 µm).

The films were also analyzed by SEM with the purpose of better understanding their surface structure. Figure 4 shows SEM micrographs of BF and JR19F.

The micrographs illustrate a porous surface on both BF and JR19F. Moreover, the presence of crystals, similar to those of JR19, can be observed on Figure 4B,C, which might be attributed to JR19, therefore corroborating the findings of optical microscopy. In Figure 4C, we can clearly see the presence of JR19 crystal entrapped in the porous structure of JR19.

The porous structure of the films may be a result of the presence of glycerol as previous reports have shown that the presence of a plasticizer in polymeric films increases the gas permeability. In fact, plasticizers such as glycerol bind to the polymer backbone, increasing the mobility of the polymer chains, which decreases the density between the polymeric network, therefore facilitating the passage of gases through the films [48,49]. Ganesan [50] and Jantrawut et al. [51] demonstrated in thier studies that these characteristics can be used as a way to control the release profile of drugs from the films, which means that the degree of porosity can be modulated by the amount of plasticizer previously added in the formulation. Further studies will be conducted to investigate the influence of the type and amount of plasticizer on the release of JR19 from ACT films. 

### 3.6. Thermal Analysis by Differential Scanning Calorimetry (DSC) and Thermogravimetry and Its Derivative (TG/DTG)

**Figure 5:**
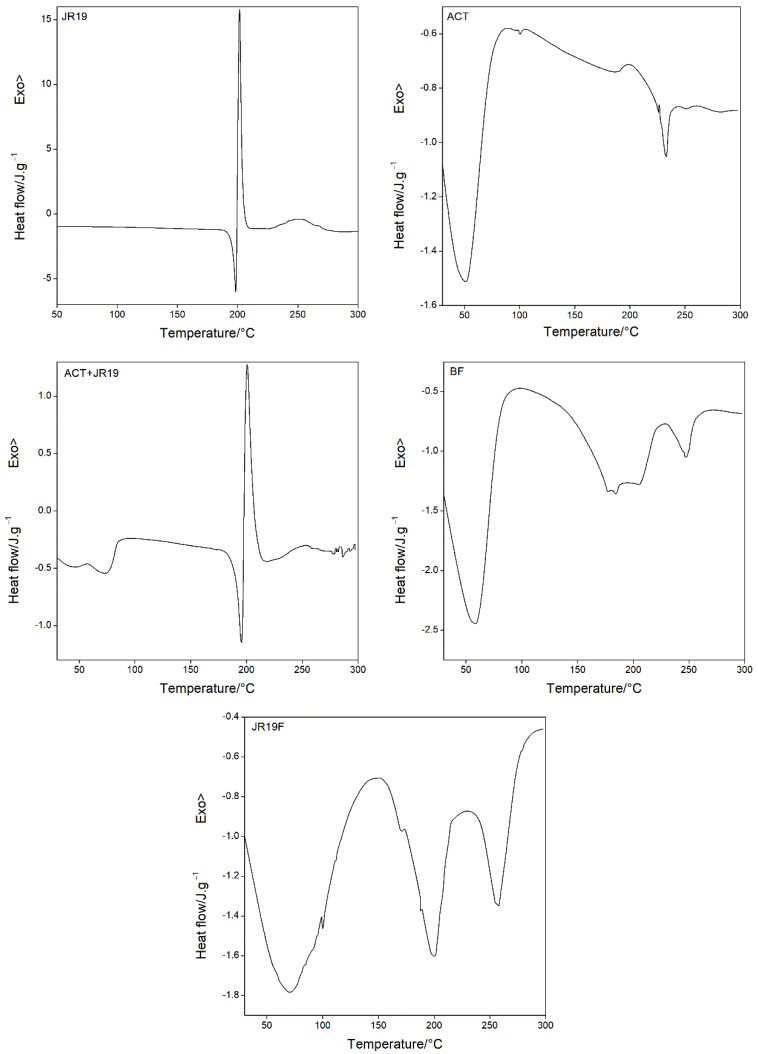
DSC curves for JR19, ACT, ACT + JR19 (physical mixture), BF and JR19F.

**Figure 6:**
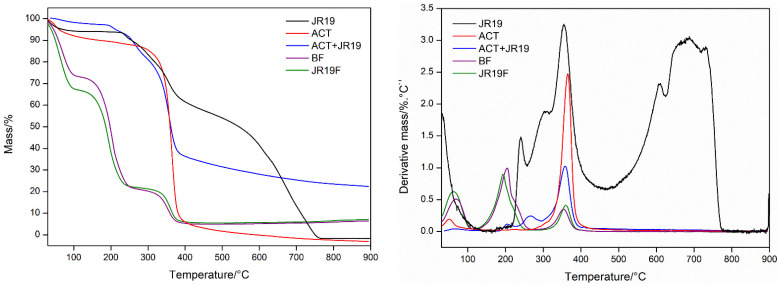
TG/DTG curves for JR19, ACT, ACT + JR19 (physical mixture), BF and JR19F.

The evaluation of the thermal behavior of each component, the physical mixture between them as well as the films is of paramount importance because it allows the investigation of possible interactions between the polymer and the drug, which might predict whether the development of the system is feasible or whether adjustments in the formulation are necessary. Figure 5 and Figure 6 show the DSC and TG/DTG curves, respectively.

The DSC curve for JR19 shows three peaks, one endothermic (Tpeak = 198.40 °C and ΔH = 41.92 J g^−1^), representing the drug’s melting temperature, and two exothermic (Tpeak = 201.64 °C and ΔH = 159.4 J g^−1^; 253.11 °C and ΔH = 59.88 J g^−1^). The DSC curve for ACT shows two endothermic peaks, the first one at Tpeak = 51.05 °C; ΔH = 185.04 J g^−1^ and the second at Tpeak = 233.00 °C, ΔH = 6.322 J g^−1^, which are attributed to the water desorption of the polymeric structure and the melting temperature, respectively. The phenomenon of water desorption can occur at different temperatures depending on the degree of substitution of cellulose acetate. Regarding the low enthalpy value of the melting temperature, the literature reports that values of this magnitude corresponds to materials with low degree of crystallinity [8,52]. The DSC curve for the physical mixture (ACT + JR19) shows two endothermic peaks, the first one at Tpeak = 75.05 °C and ΔH = 18.94 J g^−1^ corresponding to the water desorption of the polymer, whereas the second one at Tpeak = 195.61 °C and ΔH = 33.22 J g^−1^ is attributed to JR19. In addition, a third event (exothermic) is observed at Tpeak = 200.67 °C and ΔH = 55.02 J g^−1^. It is worth noting that the characteristic peaks of the melting temperature of JR19 remained unchanged in the physical mixture.

The DSC curve for BF shows a thermal profile similar to that of ACT, with two endothermic peaks, one at Tpeak = 59.09 °C and ΔH = 347.2 J g^−1^ and the other one at Tpeak = 247.66 °C and ΔH = 25.92 J g^−1^, which indicates higher thermal resistance of the films to melting and residual moisture desorption. The DSC curve for JR19F showed an increased water desorption temperature (Tpeak = 71.34 °C and ΔH = 349.6 J g^−1^) in comparison to that of ACT. A second endothermic event associated with the melting of JR19 is observed at Tpeak = 200.04 °C and ΔH = 86.50 J g^−1^, which indicates no significant variation in the melting temperature of the drug after incorporation into ACT films. Finally, a third endothermic event at Tpeak = 257.87 °C and ΔH = 47.16 J g^−1^ is observed, which indicates an increase in the melting temperature of JR19F. These findings suggest that no chemical interaction took place between the components of the films. 

Figure 6 shows that JR19 presents four stages of mass loss (30–140.71 °C, Δm = 5.97%; 140.71–253.74 °C, Δm = 3.81%; 253.74–462.4 °C, Δm = 33.51%, and 462.4–748.3 °C, Δm = 56.30%) with a residual mass of 0.41%. ACT presents two stages of mass loss (30.66–151.66 °C, Δm = 9.216%; 256.59–441.86 °C, Δm = 84.4%), the first one is due to the loss of volatile components adsorbed to the polymer or due to waste water. The second one (loss of 84.4% of ACT) is reported to be due to the thermal decomposition of ACT chains, to the loss of acetyl groups as well as due to the polymeric chain fission and carbonization, leaving a residual mass of 6.384% [53,54]. For the physical mixture ACT + JR19, the presence of four stages of weight loss is observed (39.17–110.07 °C, Δm = 2.097%; 110.07–228.23 °C, Δm = 3.545%; 228.23–295.34 °C, Δm = 11.76% and 295.34–424.85 °C, Δm = 47.08%), with a predominance of loss of mass, which is similar to the aforementioned events of ACT.

The films presented similar thermogravimetric profiles before and after the incorporation of JR19. The BF presented three stages of mass loss (30.66–117.63 °C, Δm = 25.32%; 117.63–269.82 °C, Δm = 51.81%; 284.95–413, 51 °C, Δm = 15.62%), whereas JR19F presented three stages of mass loss (29.72–114.79 °C, Δm = 30.73%; 114.79–272.66 °C, Δm = 45.05%, 272.66–432.41 °C, Δm = 16.26%). It is worth noting that a greater loss of volatile components in the films is observed on the first stage when compared to that of ACT, which seems to be due to the loss of adsorbed water and to the greater amount of volatile components in the formulation as acetone was among the solvents used to prepare the polymer solution [55]. Furthermore, a second event with a higher percentage of mass loss is observed, which seems to be due to the polymeric chain scission that gradually occurs with the increase in temperature. This process takes place simultaneously with the degradation of glycerol. In the third stage, the carbonization and decomposition of the polymer chains occurs. These findings corroborate those reported by Gonçalves et al. [5], who showed that the presence of glycerol in the formulations accelerated the mass loss of ACT films by inducing a decomposition process at lower temperatures when compared to those of ACT films without glycerol. These results demonstrate that JR19 and ACT association is viable due to the absence of incompatibility. Table 2 depicts the results of thermal decomposition and calorimetric events of the samples.

### 3.7. Fourier Transform Infrared Spectroscopy (FTIR)

**Figure 7:**
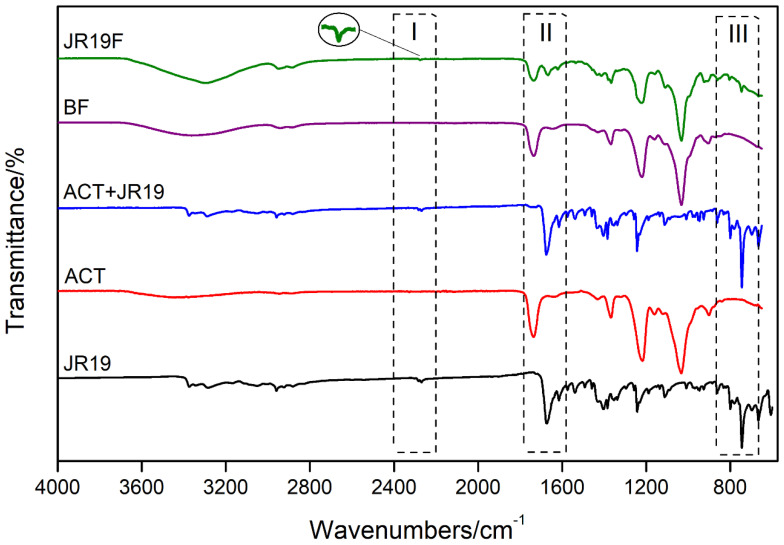
FTIR spectrum of JR19, ACT, JR19 + ACT (physical mixture), BF and JR19F.

FTIR was used as a complementary technique for the characterization of functional groups and to access any sort of chemical interactions between JR19 and ACT films. Figure 7 shows the main bands observed in the FTIR spectra.

Table 3 and Table 4 depict the main FTIR bands for ACT and JR19, respectively.

A low-intensity band at ~3480 cm^−1^ is observed in the FTIR spectrum of the films, which is attributed to O–H stretches of the cellulose acetate monomer units. According to information provided by Sigma-Aldrich^®^, this polymer presents itself as diacetate. In addition, this absorption band indicates the presence of adsorbed water. This finding is corroborated by the bands seen at around 1635 cm^−1^, which are characteristic of the angular deformation of water molecules. The bands at approximately 2940 cm^−1^ and 1730 cm^−1^ are characteristic of absorption of Csp3-H and C=O, respectively, which are attributed to the carbonyl of cellulose acetate ester. In addition, an intense band is observed at 1230 cm^−1^, which is associated with the stretching of the ester C-O bond. The bands at 1430 cm^−1^ and 1360 cm^−1^ are due to the deformation of the symmetric CH2 and asymmetric CH3 bonds, respectively, which indicate the presence of the acetylated polymer. Finally, the characteristic bands of cellulosic material are represented by the bands at 1030 cm^−1^ and 890 cm^−1^ which are attributed to the C–O bond of the cellulosic chain and to the stretching of the glycosidic bond between the glucose units of cellobiosis. It is worth mentioning that the FTIR profile of ACT was maintained for both BF and JR19F.

The FTIR spectra of JR19 show a characteristic band attributed to C≡N (2279 to 2264 cm^−1^), as previously reported by Moraes et al. [19], Pandey, Khan and Saxena [56], Ke et al. [57] and Konnola, Nair and Joseph [58]. This nitrile stretch band was also present in the FTIR spectra of physical mixture and, to a lesser extent, in that of JR19F, which suggests some interaction between JR19 and ACT as demonstrated by Wanderley et al. [59] in chitosan films entrapped with JR19.

Bands corresponding to the axial deformation of C=O of amide are observed in the region between 1677 cm^−1^ and 1636 cm^−1^. This band appears at high intensity in the spectrum of JR19 and ACT + JR19 (physical mixture); however, it appears at a lower intensity in the spectra of JR19F. Such finding might indicate that the polymer had some influence on the drug during the film formation process [59].

The absorbance band in the region of 749 cm^−1^ to 741 cm^−1^ is attributed to the angular deformation of four adjacent hydrogens (ortho-substituted aromatic rings) that come from the aromatic ring of the indole group. This band is also observed in the physical mixture and, at a lower intensity, in films with the presence of the drug (JR19F). The suppression of JR19 bands in the spectra of JR19F might be an indication of a complex formation between cellulose acetate and this drug. According to Wojnarowska et al. [60], the formation of these types of complexes between small and large molecules usually occurs through non-covalent bonds such as hydrogen bonds and Van der Waals forces.

### 3.8. X-ray Diffraction (XRD)

**Figure 8:**
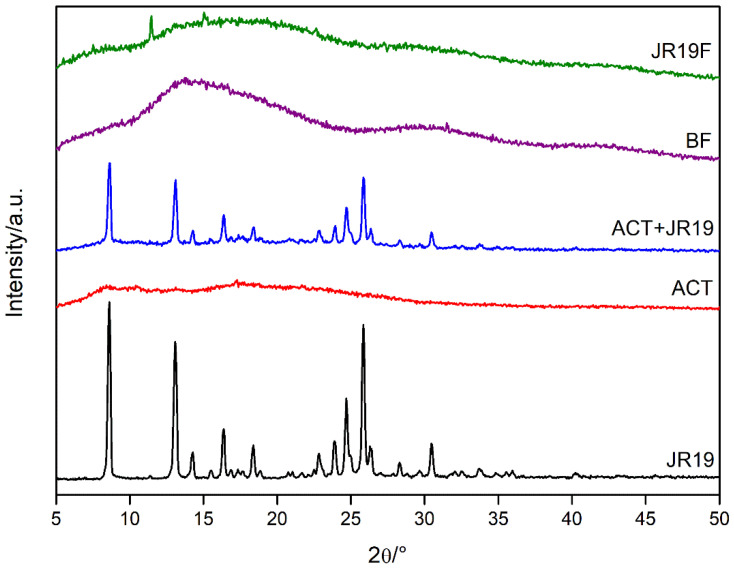
X-ray diffractograms of JR19, ACT, ACT + JR19 (physical mixture), BF and JR19F.

The XRD analyses were performed with the purpose of evaluating the degree of crystallinity and amorphicity of the samples. When X-rays interact with a crystalline material, a diffraction pattern is generated and the more three-dimensional crystalline structures are present in the materials, the more intense and narrower are the peaks in the diffractogram. On the other hand, the amorphous fraction generates broad peaks and smaller “halos” depending on the present and extent of this fraction [61]. X-ray diffractograms of JR19, ACT, ACT + JR19 (physical mixture), BF and JR19F are shown in Figure 8.

The diffractogram of JR19 shows diffraction peaks at 8.54°, 12.80°, 14.09°, 16.08°, 18.19°, 22.65°, 23.59°, 24.53°, 25.70°, 28.16° and 30.27° 2θ, characterizing the crystalline nature of this compound. For the physical mixture of ACT and JR19, the diffraction peaks of the latter appear with less intensity, which is in accordance with the findings of Wanderley et al. [59].

The diffractogram of cellulose acetate shows diffraction peaks at 8°, 10°, 13°, 17° and 22° 2θ, which are characteristic of acetylated materials with a semi-crystalline profile. The halos at 8°, 10° and 22° 2θ are considered the most important diffraction peaks, the latter being called the Van der Walls halo, which is present in most polymers due to the packaging of polymer chains as a result of the Van der Walls forces [62]. The peak at 8° 2θ is attributed to a disorder in cellulose’s structure when acetylated. Such disorder is a result of the substitution of groups along the cellulose structure, as well as due to the breakdown of its microfibrillar structure. These diffraction peaks have been attributed to cellulose acetate as previously reported by Shaikh et al. [63] and Gonçalves et al. [5].

The diffractograms of BF and JR19F are similar to that of ACT, even though the diffractogram of BF shows a decrease in the intensity of the diffraction peaks of ACT with an increase in the halo pattern. This finding indicates an amorphization of the structure, which might due to the long interatomic distances of the glycosidic bonds that link the polymer monomers making crystalline ordering impossible, therefore generating a more random structure [64]. The diffractogram of JR19F shows a decrease in crystallinity when compared to that of ACT, which suggests that the incorporation of JR19 causes a disorder in the structure of the films, thus decreasing its crystallinity. However, two peaks are shown at 12° and 16° 2θ, which corroborates with the data of the size of the crystals that indicated a decrease in their sizes after preparation of JR19F. The appearance of these peaks may be related to the presence of a new crystalline form of JR19 obtained during the preparation of JR19F that probably modified its crystalline pattern. Wanderley et al. [59] obtained similar results after the incorporation of JR19 in chitosan films. In fact, physical–chemical interactions between polymer and drug are likely to occur, which might result in the loss, decrease or change in the crystallinity of the drug. However, it can be advantageous as this process increases the solubility of the drug [65].

## 4. Conclusions

The method used in this work enabled the formation of films with reasonable macroscopic appearance and mechanical sensory. Mechanical tests showed that the films presented optimum characteristics for application as skin wound dressings. Morphological characterization showed the presence of a porous structure, which may be favorable for drug release. Finally, thermoanalytical and spectroscopic techniques allowed the evaluation of physical–chemical parameters related to interactions between JCR19 and ACT, and crystallinity/amorphism, as well as structural elucidation of functional groups, in which the results demonstrated that JCR19F is suitable for further development as a wound dressing. It seems plausible to assume that large-scale production of these films and their quality control assurance are feasible, as the techniques used are routinely employed in the pharmaceutical industry for this purpose.

## Figures and Tables

**Table 1:** Thickness, tensile strength and percentage of deformation of cellulose acetate films with and without JR19.

Sample	Thickness (mm)	Tensile Strenght (Mpa)	Deformation (%)
BF	0.132 ± 0.016	2.966 ± 0.52	5.834 ± 1.72
JR19F	0.137 ± 0.019	3.672 ± 1.45	6.556 ± 2.88

**Table 2:** Thermal decomposition and calorimetric events of for JR19, ACT, ACT + JR19 (physical mixture), BF and JR19F.

	DSC			TG		
Sample	Events	T_peak_ (°C)	ΔH (J g^−1^)	Phases	Temperature Range (°C)	Mass (%)
JR19	1 2 3	198.40 201.64 253.11	41.92 159.40 59.88	1 2 3 4	30.00–140.71 140.71–253.74 253.74–462.40 462.40–748.31	5.97 3.81 33.51 56.3
ACT	1 2	51.05 233.00	185.4 6.322	1 2	30.66–151.66 256.59–441.86	9.216 84.40
ACT:JR19	1 2 3	75.05 195.61 200.67	18.94 33.22 55.02	1 2 3 4	39.17–110.07 110.07–228.23 228.23–295.34 295.34–424.85	2.097 3.545 11.76 47.08
BF	1 2	59.09 247.66	347.2 25.92	1 2 3	30.66–117.63 117.63–269.82 284.95–413.51	25.32 51.81 15.62
JR19F	1 2 3	71.34 200.04 257.87	349.6 86.50 47.16	1 2 3	29.72–114.79 114.79–272.66 272.66–432.41	30.73 45.05 16.26

**Table 3:** Characteristic band positions and attribution of FTIR spectra of cellulose acetate (ACT).

Band Position (cm^−1^).	Attribution
3480	O–H stretch
2940	Asymmetric stretch of CH_3_
1730	Ester carbonyl stretch
1635	Water deformation
1430	Asymmetric strain of CH_2_
1360	Asymmetric strain of CH_3_
1230	Acetate C–O–O stretch
1030	C–O stretch
890	Stretching of the glycosidic bond

**Table 4:** Characteristic band positions and attribution of FTIR spectra of JR19.

Band (cm^−1^)	Attribution
3280	N-H stretch
2959	C-H stretch
2264	Nitrile stretch (C≡N)
1667	Axial strain C=O of amide
741	Aromatic ring of the indol group
